# Mangiferin-Loaded Polymeric Nanoparticles: Optical Characterization, Effect of Anti-topoisomerase I, and Cytotoxicity

**DOI:** 10.3390/cancers11121965

**Published:** 2019-12-06

**Authors:** Francisco Fabian Razura-Carmona, Alejandro Pérez-Larios, Napoleón González-Silva, Mayra Herrera-Martínez, Luis Medina-Torres, Sonia Guadalupe Sáyago-Ayerdi, Jorge Alberto Sánchez-Burgos

**Affiliations:** 1 Tecnológico Nacional de México/I.T. Tepic, Laboratorio Integran de Investigación en Alimentos, Lagos del Country, Tepic CP 63175, Nayarit, Mexico; fabianrazura@gmail.com (F.F.R.-C.); sonia.sayago@gmail.com (S.G.S.-A.); 2 Division of Agricultural Sciences and Engineering, University Center of the Altos, University of Guadalajara, Tepatitlán de Morelos CP 47620, Jalisco, Mexico; napoleon.gonzalez@cualtos.udg.mx; 3 Instituto de Farmacobiología, Universidad de la Cañada, Teotitlán de Flores Magón CP 68540, Oaxaca, Mexico; chimay_2002@hotmail.com; 4 Facultad de Química, Universidad Nacional Autónoma de México, México D.F. CP 04510, Mexico; luismt@unam.mx

**Keywords:** nanoparticles, mangiferin, anti-topoisomerase activity, cytotoxicity

## Abstract

Mangiferin is an important xanthone compound presenting various biological activities. The objective of this study was to develop, characterize physicochemical properties, and evaluate the anti-topoisomerase activity of poly(lactic-co-glycolic acid) (PLGA) nanoparticles containing mangiferin. The nanoparticles were developed by the emulsion solvent evaporation method and the optimal formulation was obtained with a response surface methodology (RSM); this formulation showed a mean size of 176.7 ± 1.021 nm with a 0.153 polydispersibility index (PDI) value, and mangiferin encapsulation efficiency was about 55%. The optimal conditions (6000 rpm, 10 min, and 300 μg of mangiferin) obtained 77% and the highest entrapment efficiency (97%). The in vitro release profile demonstrated a gradual release of mangiferin from 15 to 180 min in acidic conditions (pH 1.5). The fingerprint showed a modification in the maximum absorption wavelength of both the polymer and the mangiferin. Results of anti-toposiomerase assay showed that the optimal formulation (MG4, 25 µg/mL) had antiproliferative activity. High concentrations (2500 µg/mL) of MG4 showed non-in vitro cytotoxic effect on BEAS 2B and HEPG2. Finally, this study showed an encapsulation process with in vitro gastric digestion resistance (1.5 h) and without interfering with the metabolism of healthy cells and their biological activity.

## 1. Introduction

Mangiferin (2-C-β-Dglucopyranosyl-1,3,6,7-tetra-hydroxyxanthone) is a xanthone C-glucoside, present in several plants [1]. It is considered as a bioactive compound (BC) that has been studied for its biosynthetic and medicinal properties. In *Mangifera indica* L. tree stem in an aqueous extract the mangiferin is the major BC [2]. Despite the potential broad applications, some chemical problems have limited its clinical use; for instance, its low solubility and poor intestinal permeability [3]. About 40 mangiferin metabolites can be biotransformed in processes like deglycosylation, dihydroxylation, methylation, glucuronidation, glycosylation, and sulfatation [4]. These metabolites are the basis to consider that mangiferin can have multiple applications, overcoming the chemical limitations for its clinical use, considering different physicochemical strategies that improve its permeability and solubility [5,6,7]—since several studies indicate the power of this compounds to prevent a TNF-α and nitric oxide (NO) production [8] and down-regulating COX-2 expression [9]. However, the use of pure BC is very limited due to fast release, low solubility, poor bioavailability, as well as easy deterioration [10,11]. Therefore, to preserve the quality of a BC or to enhance its applicability in food, nutraceutical, or biological formulations, a feasible alternative has been considered, namely, nanoencapsulation. Nanoencapsulation is an important technology for the protection of bioactive compounds (BCs) [12], recently, it has focused on increasing functionalities, such as high entrapment efficiency, bioavailability, mechanical stability, controlled release, and masking undesirable flavors [13,14]. Some of the applications in the food and pharmaceutical industry seek to encapsulate BCs, with the objective of forming protective barriers that increase the specialized application in the development of nutraceuticals [15].

Generate nanoparticles (NPs) of mangiferin, a BC that has shown biological activities such as antioxidant, antihypertensive, and anti-inflammatory, will allow to increase its resistance to acidic conditions, which is related to human digestion. These NPs are encapsulated with a biocompatible polymer such as poly(lactic-co-glycolic acid) (PLGA), which can resist this process and consequently have a controlled release [13]. Thereby, the aim of this study was to develop PLGA nanoparticles containing mangiferin and to evaluate their physicochemical properties, effect cytotoxic, and the anti-topoisomerase activity.

## 2. Results and Discussion

### 2.1. Encapsulation Efficiency (EE%) and Entrapment Efficiency (AE%)

In the NP preparation, it was observed that one of the critical steps was the previous solubilization of mangiferin (MG) in polyvinyl alcohol (PVA) solution; therefore, solubility tests were performed, obtaining the maximum concentration of MG in the formulations of 435 μg/mL of PVA solution. The EE% and AE% in each treatment were obtained for each NP formulation. EE% indicates the amount of compound that is inside the NPs, and that its behavior is reflected in a gradual release with respect to time, while the AE% is the one that is in the first layers of the nanoparticles added to the surface of the particles [14].

**Figure 1:**
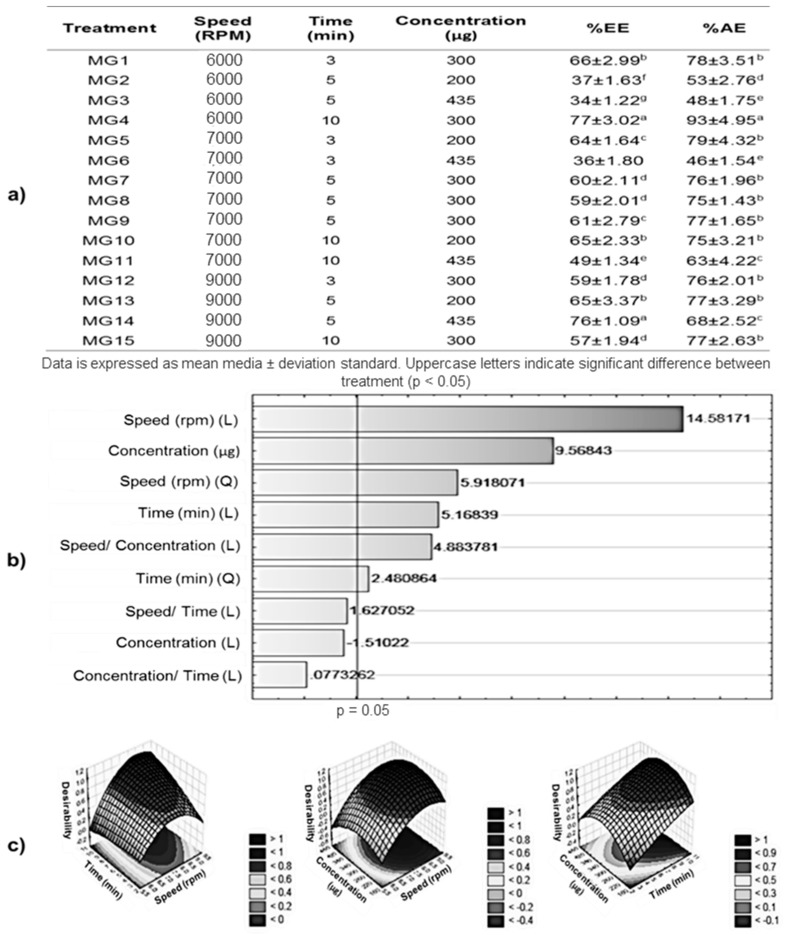
Optimization of mangiferin (MG) encapsulation. (**a**) Percentage of mangiferin encapsulation efficiency (EE%) and percentage of mangiferin entrapment efficiency (AE%) for each treatment. (**b**) Pareto chart of standardized effects; variable: Percentage of mangiferin encapsulation efficiency (EE%); (L) and (Q) describes the linear and quadratic interactions effects on the variable. (**c**) Desirability surface contours of percentage of mangiferin encapsulation efficiency; method: Spline.

In Figure 1a, the EE% and EA% corresponding to the MG formulations are shown. The treatment that presented the highest encapsulation efficiency was MG4 (6000 rpm, 10 min, 300 μg) and MG14 (9000 rpm, 5 min, 435 μg) with EE% of 77 ± 3.02% and 76 ± 1.09%, respectively; while those of lower EE% were MG3 (6000 rpm, 5 min, 435 μg) and MG6 (7000 rpm, 3 min, 435 μg) with EE% values of 34 ± 1.22% and 36 ± 1.80%. Regarding the EA%, only MG4 presented significant difference with respect to the other treatments, presenting an AE% of 93 ± 4.95%, while the lowest corresponding to MG2 (6000 rpm, 5 min, 200 μg).

Some studies reported that different polymers have shown that the EE% of some compounds is a function of the charges present between the polymer and the compound [15,16,17]. The different charges between the molecules of mangiferin and PLGA promote the interactions between both, so when the mangiferin concentration increases, the EE% increases; however, some authors have described that this increase of encapsulating compounds promotes the saturation of the system, as is observed in the MG3 and MG6 treatments, which increased the concentration of mangiferin—however, the EE% was about 36% [18]. With high stirring speeds, EE% can be increased, since there is an increase in electrostatic charges but at short times [19]; this explains why MG14 presents EE% values close to the best MG4 treatment. However, low concentrations of the compound to be encapsulated are trapped on the surface of the particles, and this behavior is observed in MG13 and MG15. According to statistical analysis (*p* < 0.5), the factor that has the greatest effect on the EE% is the speed, followed by concentration ratio and the interaction between this variables, as shown in the Pareto chart (Figure 1b); MG4 showed the highest EE% with the lowest homogenization rate studied, unlike other treatments with the same concentration but at higher speeds (MG9 and MG15). Some studies have shown that the time in which the molecules are exposed to a certain speed can be decisive, so that a molecular interaction can be exerted [20]. Therefore, the treatment with the highest EE% was formulated with the following conditions: 6000 rpm, 10 min, and 300 μg of mangiferin according to the response surface obtained in the statistical analysis (Figure 1c), which is expressed in Equation (1).

*Z* = 283.56242804966 + 62.848482862581 × *x*4.3090184425532 × *x*^22.7320158696753 × *y*.36691981479872 × *y*^2 + 1.1853803871635 × *x* × *y* +0.012224096786592 × 300 × *x* + 0.00024480875382114 × 300 × *y* + 69.8885631 (1)

where R-sqr = 0.90756, z describes EE%, *x* describes the homogenization speed, and *y* is the time at fixed concentration (300 µg).

**Figure 2:**
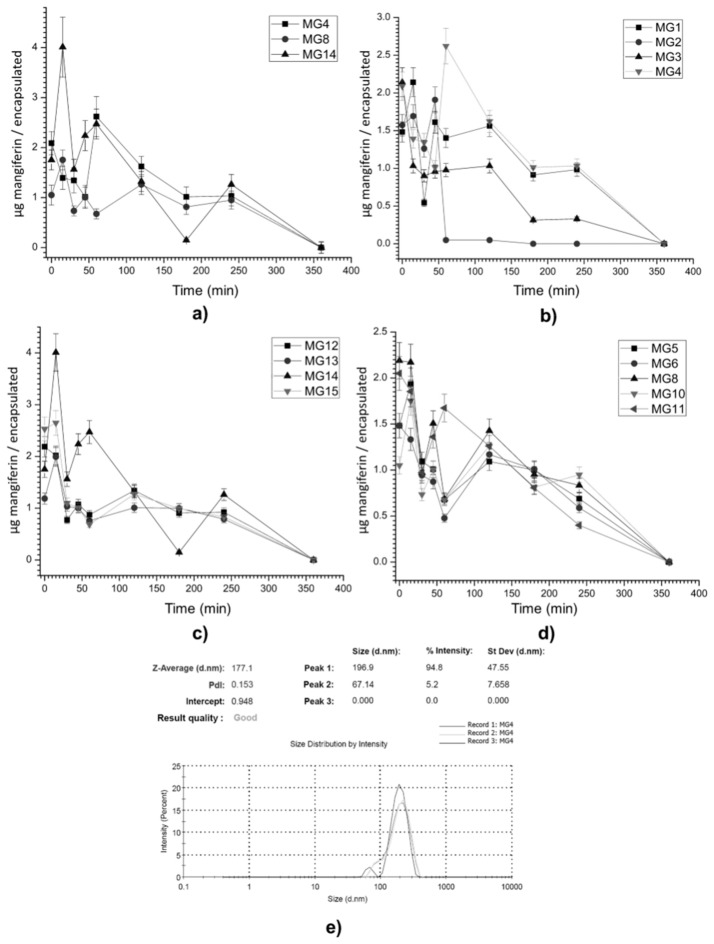
Mangiferin release profile and size distribution of best treatment; (**a**) best release profiles, (**b**) treatments at 6000 rpm, (**c**) treatments at 7000 rpm, and (**d**) treatments at 9000 rpm. (**e**) Size distribution on optimal condition (MG4).

An optimal condition was obtained when 317 µg of mangiferin was used in accordance to response surface, but this concentration can generate an increase in the size of the particles and in the polydispersity index. The size obtained in MG4 was 171 nm with a polydispersity index of 0.153 (Figure 2e).

### 2.2. Mangiferin Release Profile

The results of the kinetic release assay are described in Figure 2, where the release profiles of each encapsulated mangiferin formulation are shown (Figure 2a–d), includeing the polydispersibility index (PDI) and the average size of the resulting particle as an optimal treatment (Figure 2e). MG2 (Figure 2a) shows a rapid release, which coincides with the results of AE% for this treatment, since the highest concentration of mangiferin is found on the surface of the NPs; MG1 and MG3 (Figure 2a) show similar release profiles, since the release presents with variations without significant differences after 60 min. Figure 2c shows the treatments obtained at 7000 rpm, observing that there is no significant difference in the release after 60 min in the MG12, MG13, and MG15 treatments. Finally, in Figure 2d, it is observed that the differences between the release profiles are related to the concentrations of mangiferin used in the treatments (MG5, MG6, MG10, and MG11), observing that at long times of homogenization, EE% and EA% trend to be of the same values, which is reflected in a release of 50% before 60 min and then in three intervals (120, 180, and 240 min), with a release of 16.6% by interval.

Three fluctuations can be identified; the first occurs at 15 min, the second at 60 min, and the last after 2 h, almost in all the treatments. Nevertheless, some treatments such as MG4 (Figure 2a), MG14 (Figure 2c), and MG8 (Figure 2d) showed the highest mangiferin fluctuation after 1 h of exposure in an acid medium.

These treatments are shown in Figure 2b, which shows that MG14 presents its maximum release at 20 min and then presents a controlled release, while MG4 presents its maximum release at 60 min and subsequently shows a linear release until 180 min, without significant differences between 180 and 240 min. Studies carried out with the same phytochemical, but with a different polymer, showed a behavior like that shown in this study; this irregular behavior (ascending, descending, and ascending) was attributed mainly to the interactions between the present molecules (electrostatic interactions and hydrogen bridges) and the diffusivity of the nanoparticles [21,22]. Another result showed that when PLGA was used as an encapsulating agent, but under other encapsulation conditions, a similar diffusivity was observed [23]. During the first phase of release, agglomeration of nanoparticles occurs, and this strongly depends on the particle size, further affecting the release of the drug (amount and rate of release) from the nanoparticles.

The PLGA might have a higher tendency to agglomerate due to smaller sizes, and the release of the drug was also higher from these particles compared to other polymers. Among the factors that affect drug release, particle size is very important. We know that the particles with smaller sizes can degrade faster due to the increased surface area to volume ratio, and this might be a reason for the faster release of the mangiferin from PLGA particles in some treatments, but it is shown that at least three of the treatments (MG4, MG8, and MG14) show a controlled release in acidic conditions, maintaining the highest amount of MG and releasing it completely after 2 h of acidic exposure, which sets a pattern for the emulation of the gastrointestinal tract [24].

### 2.3. Scanning Electron Microscopy (SEM)

**Figure 3:**
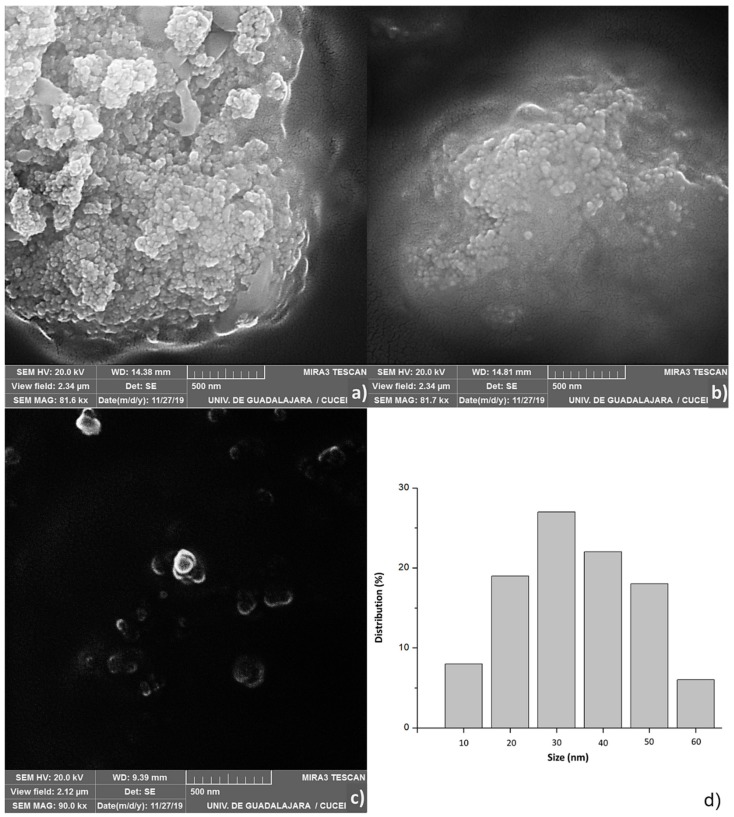
Scanning electron microscopy (SEM) of the mangiferin optimal nanoparticles (MG4); (**a**,**b**) images of optimal treatment; (**c**) control PLGA whitout mangiferin; (**d**) Pareto diagram of size distribution of optimal treatment.

Figure 3 shows, scanning electron microscopy images of the optimal treatment (Figure 3a,b, MG4; Figure 3c, control). In the images related to MG4, agglomerations of nanoparticles can be seen, which were counted 100 particles independents, and Figure 3d shows the Pareto diagram of the size distribution of NPs. Previous studies show a similar behavior in agglomerated nanoparticles of the same polymer loaded with catechins larger than 500 nm; however, there is evidence that nanometer sizes are achieved with PLGA-loaded gold [25,26]. The results shown by SEM coincide with peak two of Figure 2e, which shows an average size of 67.14. It is possible that the dispersion of the particles is not adequate, and therefore, the distributions in that technique were greater, thus quantifying conglomerates of smaller particles.

Moreover, it has been described that the chemical structure of mangiferin creates a xanthone framework made up of four phenolic units and a glucose moiety [27]. Hence, it may be assumed that mangiferin might exhibit high affinity by PLGA and PVA forming a long structure that occurs for interaction of OH groups in the chemical composition [28]. In addition, the phenolic and glucose units present in mangiferin can efficiently stabilize the formed nanoparticle. Despite its bio-relevance and strong reducing capability, the use of mangiferin is not limited towards the preparation of nanoparticles [29].

### 2.4. UV-Visible Spectroscopy

**Figure 4:**
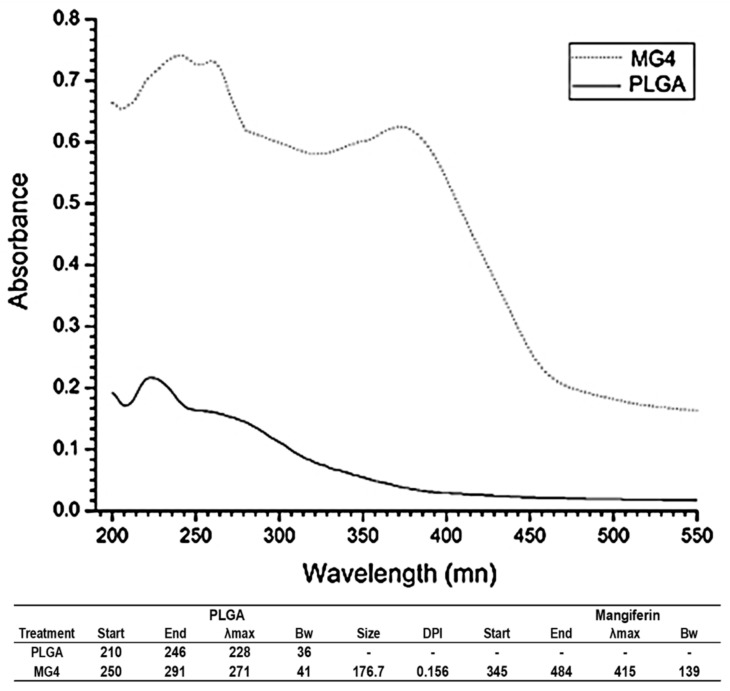
Fingerprint UV-vis of mangiferin optimal nanoparticles (MG4) and poly (lactic-co-glycolic acid) (PLGA).

The spectra obtained and reported in Figure 4 represents the optimal treatment (MG4) and only PLGA; this is related to the table that describes the same figure, in which the MG4 treatment is identified in two signals traveling to the infrared spectral region. The wavelength of greater absorption (λmax) in the PLGA-only was identified at 220 nm; this value was obtained for other authors [30]. The λmax for MG was 415 nm; according to this result, mangiferin slightly modified its orientation towards a different value UV signal. Atoms and molecules only absorb and emit radiation of certain frequencies, which implies the quantization of their energy levels. The electronic levels of a molecule are widely separated and usually only the absorption of a high energy photon can excite a molecule. UV spectroscopy studies the absorption of visible ultraviolet radiation by a molecule. By influencing UV-visible radiation of adequate energy, the molecules pass from the ground state to a state of higher energy (excited). If energy of the radiation matches the energy difference between the last occupied state and the first empty state, the transition from an electron to a higher energy state occurs. Therefore, a molecule absorbs the excitation of its busy orbital of higher energy (HOMO) to one occupied by a single electron (SOMO), according to the molecular orbital theory [31]. Some molecules have the ability to transfer between 1 to 3 electrons in this way, generating interactions and structural–energetic modifications which occur in the orbitals of each molecule; when this change take place is very likely that a molecule absorbs energy of certain wavelength modifying its spectral area [32,33,34]. Although it is not a specific method for identification of link vibration, it is possible to elucidate whether the formation of a new complex exists, or simply molecules are found. Contrary to the functionalized treatments where a modification of the spectral area of each component is shown, as there are interactions between polymeric matrix and phytochemicals, resistance of these occurs in an aqueous system.

### 2.5. Powder X-ray Diffraction (XRD)

**Figure 5:**
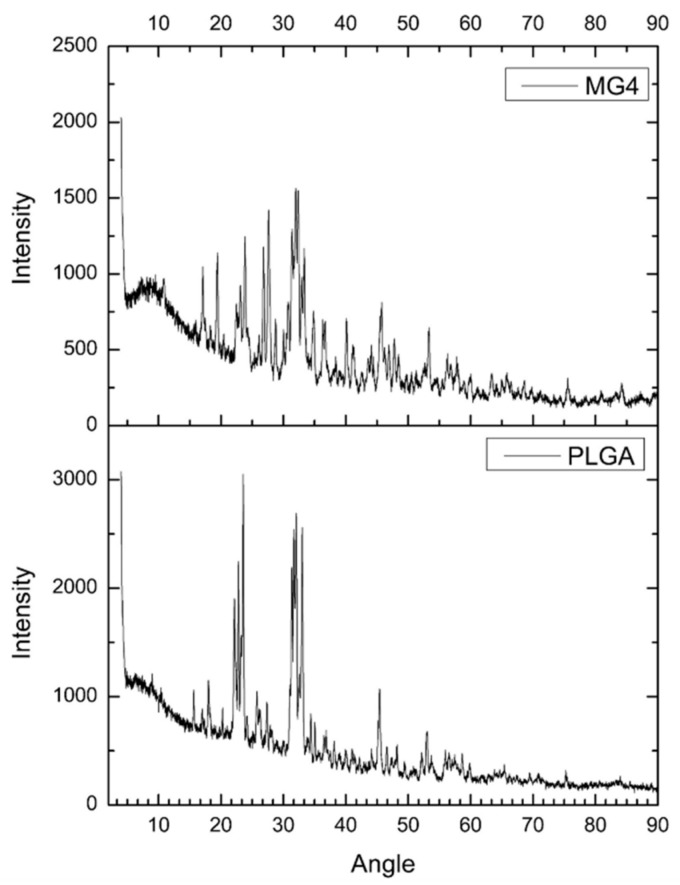
Powder X-ray diffraction patterns of mangiferin optimal nanoparticles (MG4) and poly (lactic-co-glycolic acid) (PLGA).

The powder X-ray diffraction patterns of MG and PLGA are illustrated in Figure 5. PLGA was in a crystalline form; however, in contrast, the XRD of the MG4 shows amplified signals corresponding to mangiferin between the θ 10–30 already described by other authors, indicating the change from a highly crystalline nature to an amorphous state of the complex mangiferin–PLGA [35,36]. However, these morphologies can also be attributed to the remnants of phosphate salts present in the formulation [36].

### 2.6. Anti-Topoisomerase Activity

It has been shown that during the development of a carcinoma, there is an increase in topoisomerases, because these enzymes are involved in cell replication [37]. Previous studies confirmed that mangiferin is capable of inhibiting the topoisomerase I enzyme (Topo I), involved in the splitting of DNA during cell division [38], Therefore, for this study, it was considered to evaluate its activity on Topo I in order to evaluate if there is loss of biological power or if it can be increased due to the controlled release of MG. Due to this, the used JN394 genetically modified strain (Matα ura3-52, leu2, trp1, his7, ade1-2, ISE2, rad52::LEU2) promotes a deficiency in the regeneration of DNA, greater permeability in the cell membrane y JN362a (Matα, ura3–52, leu2, trp1, his7, ade1–2, ISE2) resistant to DNA repair but sensitive to antimicrobial agents.

**Figure 6:**
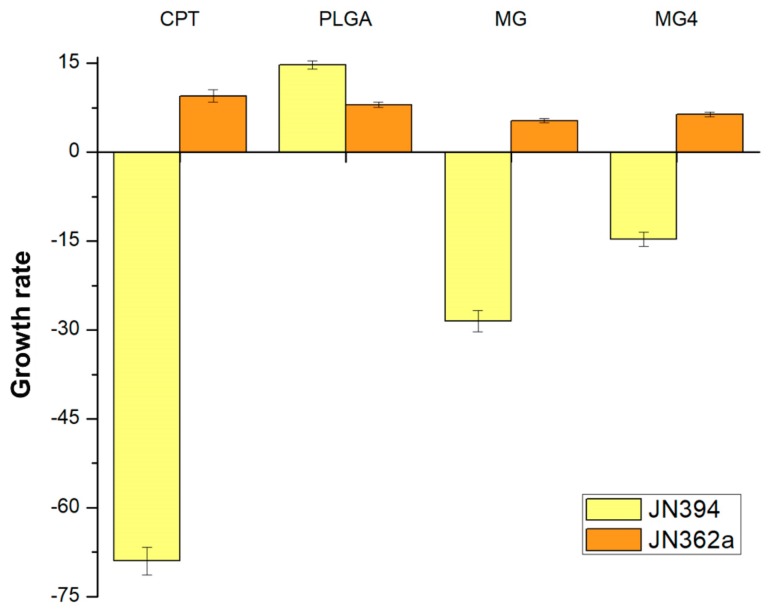
Growth rate of Camptotecin (CPT), poly (lactic-co-glycolic acid) (PLGA), mangiferin (MG), and mangiferin nanoparticles (MG4) in the proliferation of modified strains of *Saccharomyces cereviseae* JN362a and JN394.

The concentration of extracts used in the assay was based on the solubility factor for each solid extract in dimethylsulfoxide (DMSO). As shown in Figure 6, the strain JN394 was hypersensitive to camptotecin (CPT) (69 ± 2.3% inhibition), which is a Topo I poison. MG4 showed 14.71 ± 1.2% inhibition (14.28 µg mangiferin/mg encapsulated) in this strain, while MG without encapsulation showed a percentage of inhibition of 28.5 ± 1.8% at the same concentration as CPT (50 μg/mL). The evaluated concentration of MG in MG4 is 5.71 times higher than that used in the camptothecin; so, to have a 69% inhibition, 66.98 μg of mangiferin (4.69 mg/mL of nanoparticles) is required.

JN394 is a strain that is DNA repair-deficient and drug-permeable (carry ise2 and rad52 mutations) [39]. These mutations increase the sensitivity of these cells to drugs [40]. The yeast JN362a, a DNA repair-proficient strain [35], was not affected by any NP treatment (+8 ± 0.43%) PLGA and (+5.3 ± 0.21%) MG4. These results mean that the MG4 has compounds with anti-topoisomerase activity against Topo I. Mangiferin has been identified as an inhibitor of the enzyme topoisomerase I by other authors [41].

The difference observed with respect to inhibition is related to the controlled release of MG4, since there is a time difference of 45 min, so that the total concentration of the encapsulated MG can be released in the media grown while CPT and MG come in contact with the yeasts from time 0 of growth. However, it has been shown that encapsulation can ensure that the amount of compound released can have a direct effect, without undergoing possible alterations during the process to reach the target cells. Mangiferin studies at a concentration of 50 µM have demonstrated an antiproliferative effect on cancer cells without affecting healthy cells; this is due to the activation of Nrf2–ARE signaling cascades [42], the controlled release of the phytochemical contributes to the inhibition of the topoisomerase enzyme, when mangiferin is expelled during the different phases of the cellular reproduction of the yeast, promoting cell death due to encapsulation [43]. A similar behavior was observed in the encapsulation of topotecan, which is a selective topoisomerase II inhibitor. A difference in cell viability between the encapsulated and non-encapsulated compound was observed—when the compound was encapsulated, the cytotoxicity remains constant after 24 h, whereas the non-encapsulated compound tended to decrease, confirming a controlled released on cell proliferation inhibition [44]. Modify strains from *Saccharomyces cerevisiae* have been used in cytotoxicity studies against topoisomerases, which were found in greater proportion in cancer cells, in addition to the modification in the gene that codes for topoisomerase [45], and these strains were like those used in the present study.

### 2.7. Cell Viability

The NPs may have a high risk on human health, and to evaluate this, different types of cell cultures have been used as toxicity models under in vitro condition. HEPG2 derivate from hepatocellular carcinoma is a cell line well established and widely used as a model for drug metabolism and cytotoxicity studies, because these cells display many features of normal liver cells [46]. BEAS2B is an immortalized cell line isolated from normal human bronchial epithelium, and it has been employed to evaluate in vitro toxicity of some nanomaterials, because it is a non-cancerous epithelial cell type [47,48]. Thus, we selected HEPG2 and BEAS2B cells as a model system for studying the in vitro toxic effects of MG4.

**Figure 7:**
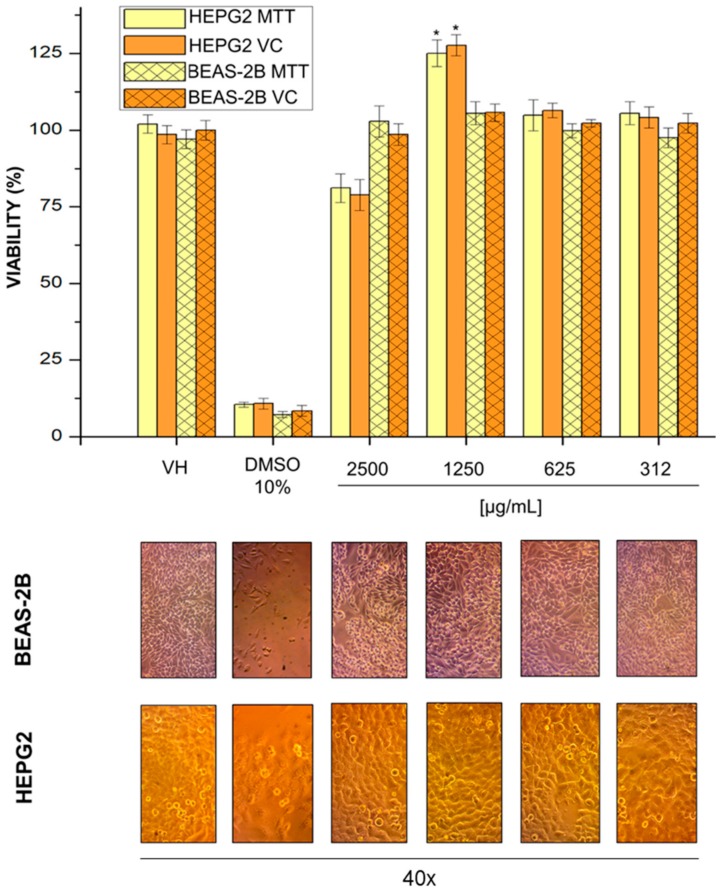
Cell viability of the various mangiferin nanoparticle concentrations for HepG2 and BEAS-2B cells using the violet crystal and MTT assay. * *p* ˂ 0.05, statistical difference between treatment and control.

HEPG2 and BEAS-2B cells were treated with varying concentrations of MG4. MTT is a tetrazolium salt that is converted to formazan salt (blue color) by mitochondrial dehydrogenase enzymes; thus, color can be measured, and it correlated with cell metabolic activity or live cells. Violet crystal dye stains cell proteins and DNA, and the cells that suffer cell death lose their adhesion; thus, adherent cells can be stained, and color can be quantified. Both methods showed that MG4 high concentrations (2500 µg/mL) were not able to decrease cell viability in BEAS-2B or HEPG2 cells (Figure 7). Furthermore, MG4 did not alter cell morphology in either cell line. Reports have shown that glycosylated bioactive compounds do not have an hepatotoxic effect on the in vitro model. Furthermore, the mangiferin conjugated with other similar compounds could have a positive role on hepatic glucose metabolism [44], as well as studies on encapsulation with PLGA showing that it does not appear to have an hepatotoxic effect on HEPG2, and therefore, it has been cataloged as a safe biopolymer [49].

Particularly, at 1250 mg/mL, an increase in the cell viability of HEPG2 is shown compared to the negative control. Other studies with this cell line have shown that some plant phenol extracts promote cell growth, but with isolated phenolic compounds, and the viability in healthy liver cells decreases at concentrations greater than 2 mg/mL [50,51]. Therefore, it is likely that this concentration allows the cellular replication of healthy cells; nevertheless, studies in cancer cells to corroborate this hypothesis are lacking.

## 3. Materials and Methods

### 3.1. Reactives

Poly (lactic-co-glycolic acid) (PLGA), 75:25, Mw 25,000; polyvinyl alcohol (PVA) Mw 85,000–124,000, mangiferin, and dicloromethane (DCM) were obtained from Sigma-Aldrich (St. Louis, MO 63118, USA).

### 3.2. Preparation of Nanoparticles (NPs)

The NPs were developed following the method of solvent evaporation [52] using 15 mg of PLGA (75:25) and adding 500 µL of DCM in a flask. The aqueous phase composition was 5 mL of PVA solution (0.5%), and the quantity of mangiferin was added according to the amount required (Figure 1). The emulsion was sonicated for 5 min. Samples were homogenized by an Ultra-turrax^®^ (IKA, T18; Germany) disperser and the organic phase was added drop-by-drop. Organic solvent was separated by rotoevaporation (Buchi, R-300; Essen, Germany). After, NPs were kept for 2 h at −80 °C and freeze-dried at −50 °C in a freeze-dryer (Labconco, FreeZone 6; Kansas, MO, USA). Finally, the lyophilized samples were stored in a desiccator and placed in the freezer (−20 °C) (Torrey, CHTC-255; Monterrey, Nuevo León, México). Loaded NPs were named MG1 to MG15, depending on the processing conditions. Unloaded NPs (PLGA) were also prepared and used as control.

### 3.3. Experimental Design

According to Box–Behnken design, a total number of 15 experiments, including 12 factorial points at the midpoints of the edges of the process space and three replicates at the center point for estimation of pure error sum of squares, were performed to choose the best model among the linear, two-factor interaction model and quadratic model due to the analysis of variance (ANOVA). An obtained *p*-value less than 0.05 was considered statistically significant. The selected independent variables were speed (A), time (B), and concentration (C) at three different levels as low (−1), medium (0), and high (+1). Dependent variables were encapsulation efficiency (EE%) and entrapment efficiency (AE%). The coded factors and responses of the variables are given in Figure 1.

### 3.4. Evaluation of Mangiferin Encapsulation Efficiency (EE%) and Entrapment Efficiency (AE%)

The samples were dissolved in phosphate-buffered solution (PBS) at pH of 7.0 solution and mangiferin AE% was determined indirectly [53]. An aliquot (200 µL) of sample was placed in a microplate reader (Biotek, Synergy HT; Winooski, VT, USA), the reading was recorded at 365 nm, and the concentration was obtained by a calibration curve of manguiferin (0.4, 0.8, 1.6, 3.2, and 6.4 µg/mL) using Equation (2).

(2) $$AE\%\;=\;\left.\left(\frac{A1-AA\;}{A1}\right.\right)\times 100$$

where *A*1 is the initial amount of mangiferin, and *AA* is the amount of free no-entrapped mangiferin determined by UV-vis [54].

*EE*% was determined later to expose the NPs under the conditions mentioned above; nevertheless, for this analysis, aliquots were taken at the time 0.15 min and 24 h. *EE*% was obtained using Equation (3).

(3) $$EE\%\;=\;\left.\left(\frac{E1-E24\;}{E1}\right.\right)\times 100$$

*E*1 is the difference in time concentration from t0 to 15 min, *E*24 is the total concentration released at 24 h. With this model it can wash the surface of the NPs with the objective of obtaining the concentration retained inside the particle.

#### Optimization of Data Using Response Surface Methodology (RSM)

Optimization by RSM was based on the highest possible value of EE% and AE% that we evaluated in terms of statistically significant coefficients and R^2^ values. A Pareto chart was used for identification the quadratic and lineal effects from independent variables.

### 3.5. Mangiferin Release Profile in NPs

The in vitro release profile of mangiferin from NPs was evaluated suspending a 1.0 mg of NPs into 5 mL of PBS solution at different pH value (1.13 and 7.05) [55]. The suspension was maintained at 37 °C and 150 rpm (magnetic stirrers). The samples were read at 0, 15, 30, 45, 60, 120, 180, 240, and 360 min. The kinetic analyses of the release data were performed using various mathematical models [56,57,58]. From the optimum condition, particle size, size distribution, and the physicochemical properties were evaluated.

### 3.6. Optical Characterization

#### 3.6.1. Size Distribution of Mangiferin NPs

The size distribution was determined in pure water at 18 °C using a particle size analyzer (Malvern, Mastersizer 2000; UK). For the measurements, 200 mL of the NP suspension was dispersed in 2 mL of filtered water. The analysis was performed at a scattering angle of 90°, refractive index of 1.590 (corresponding to PLGA), and 18 ± 3 °C.

#### 3.6.2. Evaluation Morphology by Scanning Electron Microscopy (SEM)

The NPs were observed under scanning electron microscope (Tescan, MIRA3 LMU, London, UK). The samples were sputter-coated with gold before observation under SEM. Both low and high magnification images were obtained to confirm the uniformity of the particle sizes and to determine the exact size of the particle, respectively. The high magnification SEM images were interpreted by ImageJ software to determine the size of the particles.

#### 3.6.3. UV-Visible Spectroscopy

UV–visible spectrums of NPs were recorded from 300 to 600 nm. Particle size distribution was carried out by a Dynamic Light Scattering (DLS) analyzer (Shimadzu, UV- 26000; Kyoto, Japan).

#### 3.6.4. Surface Composition of the Np´s by X-ray Diffraction (XRD)

The XRD patterns were obtained using a Bruker D8 Advance equipment diffractometer (Tokyo, Japan) (k = 1.5460 Å, 40 KV, 30 mA), The diffraction intensity as a function of the diffraction angle (2θ) was measured between 10 and 90°, using a step of 0.02° and counting time of 0.25 s per step.

### 3.7. Biological Material

Mutant yeasts of *Saccharomyces cerevisiae*, JN362a and JN394 cells, were donated by Dr. John Nitiss of St. Jude Children’s Research Hospital, Memphis, Tennessee. Cell lines were obtained of American Type Culture Collection: Primary and immortalized human bronchial epithelial cells, BEAS-2B (ATCC CRL-9609), and hepatocarcinoma adherent epithelial cell, HEPG2 (ATCC HB-8065).

### 3.8. Yeast Anti-Topoisomerase Assay

The anti-topoisomerase activity was evaluated using mutants *S. cerevisiae* JN362a and JN394 strains [59]. Briefly, yeast cells were grown in YPDA media at 30 °C for 18 h in a shaking incubator. The logarithmically growing cells were then counted using a hemocytometer and adjusted to a concentration of 2 × 10^6^ cells/mL media. Yeast cells (6 × 10^6^ cells) were incubated at 30 °C for 24 h in the shaking incubator (Thermo scientific, SHKE4450; Bedford, MA, USA) in the presence of the NPs, mangiferin, or CPT previously dissolved in 50 µL DMSO. DMSO (1.66%) was used as negative control, while CPT (50 µg/mL), a topoisomerase I inhibitor, was the positive control. Treated cells from each mixture were then duplicate plated to petri dishes containing 1.75% Agar bacto solidified YPDA media. Cells were incubated at growth temperature of 30 or 25 °C for 48 h. The anti-topoisomerase activity was determined as number of counted colonies in each plate by comparing to that of the negative control (DMSO).

### 3.9. Cell Line Culture

The cell lines BEAS-2B and HEPG2 were cultured in Dulbeco’s Modified Eagle’s Medium (Gibco 12320-032 and 12100-038, respectively; Gaithersburg, MD, USA) supplemented with 10% fetal bovine serum (JRScientific Inc., 43640-500; Woodland, CA, USA) and 1% streptomycin/penicillin (PAA, P11-002). Cultures were maintained at 37 °C in 5% CO_2_.

### 3.10. Cell Viability Assay

Cell lines were seeded (1 × 10^4^ cells/100 µL/well) in 96-well plates for 24 h in complete media [60]. Then, cells were treated with MG4 in concentrations of 2500, 1250, 625, and 312 µL/mL, medium volume was completed at 200 µL, and cells were incubated by 72 h at 37 °C in 5% CO_2_. The DMSO (at the same volume that NPs, 1%) was used as a negative control and DMSO to high concentration (10%) was used as a positive control. Citotoxicity was evaluated by violet crystal and 3-(4,5-dimethylthiazol-2-yl)-2,5-diphenyl tetrazolium bromide (MTT), where all experiments were performed in triplicate in three independent experiments; thus, all data are reported as the mean value ± the standard error of the mean. The cells were observed with an inverted optical microscope (Olympus, CKX-41; Waltham, MA, USA.) and photographs were taken with the Microscope Eyepiece camera (AmScope, MU130; Irvine, CA, USA).

#### 3.10.1. Staining with Violet Crystal

After 72 h of exposure to the NPs, cells were fixed with p-formaldehyde 4% (75 µL each well), and incubated at 37 °C for 1 h. Next, three washes were performed with PBS, and the plate was inverted and dried for 2 h in absorbent paper. Then, violet crystal 0.5% (50 µL to each well) was added and incubated at 37 °C for 20 min. Each well was washed three times with PBS, and subsequently, it was dried for 24 h. Finally, methanol (200 µL) was added to each well, and the plate was shaken and read at 570 nm in a BioRad microplate reader (iMark™ Microplate Absorbance Reader). The cell viability percentage was calculated as a ratio to the values obtained by the untreated cells. The results were analyzed with GraphPad Prism 5.0 software.

#### 3.10.2. MTT Tetrazolium Assay

The cell cytotoxicity was evaluated using MTT stock solution (0.5 mg/mL); for that, MTT was dissolved in phosphate-buffered saline (PBS) (pH 7.4), and then it was filtered and stored at −20 °C in the absence of light. The assay was performed according to [61]; briefly, cells treated with NPs were washed once with PBS, then MTT (50 µL) and PBS (50 µL) were added to each well and incubated for 4 h at 37 °C in 5% CO_2_. MTT solution was removed, and 100 μL DMSO was added to each well to dissolve the formazan crystals. Then, the plate was shaken and read at 570 nm in a BioRad microplate reader (iMark™ Microplate Absorbance Reader). The cell viability percentage was obtained by comparing the results with those of untreated cells. The results were analyzed with GraphPad Prism 5.0 software.

## 4. Conclusions

The nanoparticles made under the solvent emulsion and evaporation method with PLGA are potentially resistant to acidic conditions for up to 45 min; the best encapsulation and entrapment efficiency (77% and 93%) was achieved at a concentration of mangiferin of 300 μg. It was observed that the three factors studied (concentration, time, and speed of homogenization) affect the efficiency of encapsulation and modify the release profile of mangiferin. Interactions between the molecules in the formulation affected the fingerprint of the compounds when encapsulated; however, this formation of bonds does not produce a negative effect on the antipopoisomerase activity of mangiferin and does not present hepatotoxicity in vitro.
